# The application of deep neural networks to the detection of lumbar hernias: results from the approaches based on convolutional networks and transformers

**DOI:** 10.3389/fradi.2026.1722312

**Published:** 2026-09-18

**Authors:** António Fernandes, João Pedro Mazuco Rodriguez, Susana Moleirinho, Irina Trofimenko, Ekaterina Guseva, Alexander Martinovich, Ilzane Maria de Oliveira Morais, Louise Bisolo, Luís Mendes Gomes, José Machado

**Affiliations:** 1 SliceD Group Research Center, Lisbon, Portugal; 2 ALGORITMI, LASI, Universidade do Minho, Braga, Portugal; 3 Faculdade de Ciências e Tecnologia, Universidade dos Açores, Ponta Delgada, Portugal

**Keywords:** 3D classification models, deep learning, disc herniation, lumbar spine MRI, medical image segmentation, radiology automation

## Abstract

**Background:**

The increasing demand for lumbar spine MRI exams, combined with radiologist shortages, has underscored the need for automated tools to assist in diagnosing degenerative spinal conditions. Despite the proliferation of AI tools in radiology, no CE or FDA-approved applications currently focus on pathologies in the lumbar spine. This study aimed to develop a pipeline for automatic identification of intervertebral disc herniation, contributing to more efficient clinical decision-making.

**Methods:**

A dataset of 484 lumbar spine MRI examinations acquired from four manufacturers was used to develop a multi-stage deep learning pipeline. An overview of the proposed automated lumbar spine MRI analysis framework is presented, illustrating the main stages of the pipeline including dataset preparation, disc segmentation, feature extraction, and classification. A subset of 165 studies (5,200 sagittal slices) was manually annotated by radiologists and used for training and validation of vertebrae and intervertebral disc segmentation models, including U-Net, DeepLabV3+, and MA-Net with EfficientNet-B2 backbones. Anatomically informed post-processing was applied to assign disc-level labels and extract disc-centered volumes. Disc-level binary classification (herniated vs. non-herniated) was then performed using 3D patch-based models, including ResNet, SENet, EfficientNet-B2, and Vision Transformer (ViT) architectures, with all dataset splits performed at the patient level.

**Results:**

The best segmentation performance was achieved by MA-Net with EfficientNet-B2, yielding a Dice Score of 0.898 for three-class segmentation (background, vertebrae, discs). For classification, the evaluated models achieved F1-scores between 0.66 and 0.73. The best performance was obtained with a ResNet18-based model, indicating that once anatomical localization and disc extraction are addressed by the segmentation stage, lower-capacity architectures can be effective for binary classification. The simplified three-class segmentation improved model robustness, and post-processing enabled disc-level precision essential for downstream classification, which achieved consistent performance across models under the available data regime.

**Conclusion:**

The proposed AI pipeline successfully automates the identification of lumbar disc herniation in MRI exams. This approach has the potential to enhance diagnostic efficiency, reduce radiologist workload, and improve reproducibility in identifying disc pathologies. While limited by sample size and class imbalance, the methodology lays a strong foundation for scalable annotation and integration of AI in routine spinal diagnostics.

## 1 Introduction

Radiology is one of the key pillars of modern medicine, providing visual and quantitative data on human morphology, function, and even metabolism. It is dedicated to diagnosis and subsequent therapy based on imaging technology. However, the increasing demand for imaging examinations far exceeds the availability of radiologists. In many imaging centres, the time and effort required cannot be adequately allocated to the analysis of medical images. The exponential growth in radiological report requests has resulted, on the one hand, in a decline in the ability to analyse all cases with due rigor, leading to diagnostic errors and reports with insufficient information. On the other hand, this situation causes delays and errors in proposing treatments, which is detrimental to patients (1, 2). Automation is key in this regard, as it can alleviate radiologists' workload and reduce errors caused by fatigue and the high volume of radiological reports (3).

Artificial intelligence (AI) techniques have gained significant momentum in various medical science fields, and radiology is no exception. Currently, 223 AI applications in radiology have been approved by the CE or FDA, with 46 of them focused on neuroradiology, according to the updated database from Grand Challenge (4). None of these applications, however, evaluate pathologies in the lumbar spine region (4).

Low back pain is one of the most common forms of disability among patients due to various causes. Lumbar spine magnetic resonance imaging (MRI) represents the most frequently requested examination for diagnostic determination (5, 6).

These challenges make lumbar spine MRI one of the most beneficial areas for developing an application that can automatically stratify patients based on pain sources and treatment needs. In a standard lumbar MRI examination, the radiologist analyses at least five-disc levels, with five spatial locations at each level: central, both paracentral regions, and both foraminal regions. Beyond these locations, the full extent of the lumbar spinal canal, the cauda equina, descending nerve roots, vertebral bodies, facet joints, ligamentum flavum, and the integrity of the posterior elements must be evaluated (7).

In patients with degenerative pathologies, many of these components are affected and must be reported individually, requiring considerable time and effort. These assessments are also largely subjective, with low interobserver and intraobserver reproducibility (8). Therefore, an application that quantifies each anatomical structure at each level would significantly assist radiologists by reducing the time needed to produce reports and increasing efficiency. This tool enables radiologists' highly specialized knowledge to be utilized more efficiently—for instance, in identifying more complex diseases rather than focusing on analysing degenerative spine pathologies, which is largely a repetitive task (9).

There is significant ambiguity in identifying the primary sources of pain in imaging examinations, which often reveal multiple degenerative changes at different levels. Finding the correlation between the true pain source and the image phenotype among various image-based candidates is an important and highly beneficial task for both patients and the specialists responsible for their treatment (10).

Several studies have investigated the application of deep learning techniques to lumbar spine MRI, including convolutional neural networks and, more recently, transformer-based architectures for disc herniation and degenerative spine assessment (11–13, 40, 41). These works have demonstrated the feasibility of automated analysis and reported promising classification and segmentation performance, contributing significantly to the advancement of AI-assisted spinal imaging.

However, despite these advances, important methodological and translational gaps remain. Many published approaches rely on slice-level or volume-level classification without explicit disc-level anatomical localization, which introduces ambiguity when multiple discs with different pathological states are present within the same examination (12, 14). In routine clinical practice, disc-level identification is essential, as radiological reporting and therapeutic decisions are made at specific vertebral levels rather than at the image or volume level.

In addition, a substantial proportion of existing studies employ homogeneous datasets acquired from single vendors or standardized acquisition protocols, limiting their generalizability to real-world clinical environments (15, 16). Scanner-related variability in image appearance and file structure remains an underrepresented challenge in lumbar spine AI research, despite its critical importance for clinical translation.

Another key limitation concerns the scalability of annotation workflows. High-quality disc-level annotations require extensive expert input, which restricts dataset size and limits the exploration of finer-grained pathological subclasses. Few studies explicitly address strategies to reduce annotation burden while preserving anatomical and pathological fidelity (9, 17).

Finally, although multiple deep learning architectures have been proposed for lumbar spine analysis, systematic comparisons between convolutional and transformer-based models under identical experimental conditions remain scarce. Variations in datasets, preprocessing pipelines, and evaluation metrics often confound direct comparisons, making it difficult to isolate the impact of architectural design choices (18–20).

To address these needs, this work aimed to develop an innovative interface for diagnosing and treating pain caused by spinal pathologies. In its initial phase, this tool will enable radiologists to make faster and more effective diagnoses of the origin of lumbosacral root compression (sciatic pain) by developing an algorithm that facilitates the identification of this pathology in a simple and intuitive manner.

We developed an advanced automated algorithm for the assessment of lumbar pathology, aiming to enhance both the efficiency and accuracy of nerve root compression detection—specifically, the presence or absence of disc herniation—by analysing images acquired through MRI of the lumbar region. This research is driven by the fundamental question: Which deep learning architecture demonstrates superior efficiency in reliably determining the presence or absence of disc herniation in lumbar spine MRI scans? We performed a rigorous comparative analysis of various architectures, evaluating their performance based on critical metrics and thereby contributing to the optimization of automated clinical assessment in spine pathology.

The present work starts with the introduction of key theoretical concepts such as compressive mechanisms of lumbar pain, particularly disc herniation, and examines the integration of artificial intelligence in radiology to enhance the automated detection of radicular compression from MRI scans, aiming to improve diagnostic accuracy and clinical decision-making. We then detail the methodology employed to train, validate, and test AI models for automated segmentation of lumbar structures and classification of disc herniation based on MRI analysis. We then examine and discuss the results through the detailed validation and testing of the developed models while exploring real-world applications in medical radiology. Finally, we summarize the study's key findings, identify its limitations, and outline potential future research directions, both methodologically and in terms of clinical applicability to lumbar spine radiology.

## 2 Methods

Initially, we explored the characteristics of the dataset used in the study, detailing its composition, sources, and relevance to the study of lumbar spine pathology. The annotation process conducted by radiologists, ensuring precise labelling of pathological features essential for training deep learning models, is presented next. Additionally, the segmentation methodology is described, highlighting the techniques and architectures employed to accurately delineate intervertebral discs and vertebrae in MRI scans.

### 2.1 Constructing the dataset

The lumbar spine MRI cases used for the construction of the dataset for the automated lumbar pathology reading algorithm were collected from scanners developed by four different manufacturers, namely: GE Healthcare, Philips, Toshiba, and Siemens. This approach aimed to maintain a considerable variety of acquisition equipment to encompass a diverse patient population and represent different acquisition systems.

The full dataset comprised 484 lumbar spine MRI studies, each corresponding to a unique patient and acquired in the sagittal plane using standard clinical protocols (e.g., T1-, T2-weighted, and STIR sequences). While all 484 MRI studies were used for classification model development, final performance metrics were computed exclusively on the fixed 100-case patient-level hold-out test set. Cases with prominent artifacts, severe scoliosis, or dominant non-degenerative pathologies—such as metastatic disease, marrow-replacing lesions, spondylodiscitis, and compression fractures—were excluded. A demographic analysis showed that 60.3% of the examinations were from male patients. The mean age was 51.54 ± 16.2 years (range: 19–82 years), with an average height of 1.757 ± 0.07 m (range: 1.60–1.90 m) and an average weight of 84.20 ± 11.69 kg (range: 60–129 kg).

Due to annotation time constraints, a subset of 165 studies was manually annotated and used for training and validation of the vertebral and intervertebral disc segmentation model, resulting in 325 sagittal series and 5,200 images. The distribution of manufacturers for this annotated subset showed a predominance of images acquired from Siemens scanners (64), followed by Philips Medical Systems (48), Toshiba (27), and GE Healthcare (26). The average image resolution was 487.1 ± 121 pixels in height and 486.35 ± 120 pixels in width, with no restrictions on sequence type, although T1- and T2-weighted images were the most common.

The classification dataset included all 484 MRI studies, comprising the 165 cases used for segmentation in addition to the remaining unannotated cases. Classification validation cases did not overlap with the segmentation training or validation sets, thereby preventing data leakage between tasks. For the complete classification dataset, the distribution of MRI scanner manufacturers was dominated by Siemens (378 cases), followed by Philips Medical Systems (48), Toshiba (32), and GE Healthcare (26). For each study, the five lumbar intervertebral discs were analyzed individually.

It should highlight the technical complexity involved in the processing and standardization of the data used. The imaging exams were acquired in DICOM format from various scanners and manufacturers, introducing significant variability both in the images and file structures. In certain cases, such as those from Philips equipment, the entire series was stored in a single.dcm file, whereas for other manufacturers, images were distributed across multiple separate files. This heterogeneity necessitated the development of specific routines for reading, standardization, and volumetric reconstruction to ensure that all samples could be uniformly processed within segmentation and classification pipelines.

Despite this variability, the pipeline (Figure 1) demonstrated robustness, adapting seamlessly to different formats without any performance loss. Additionally, tools were developed to export the segmented volumes in the.nrrd format, which is compatible with medical visualization software such as *3D Slicer*. This compatibility enabled visual validation of segmentations generated by AI as well as facilitated the manual annotation process for radiologists.

**Figure 1:**
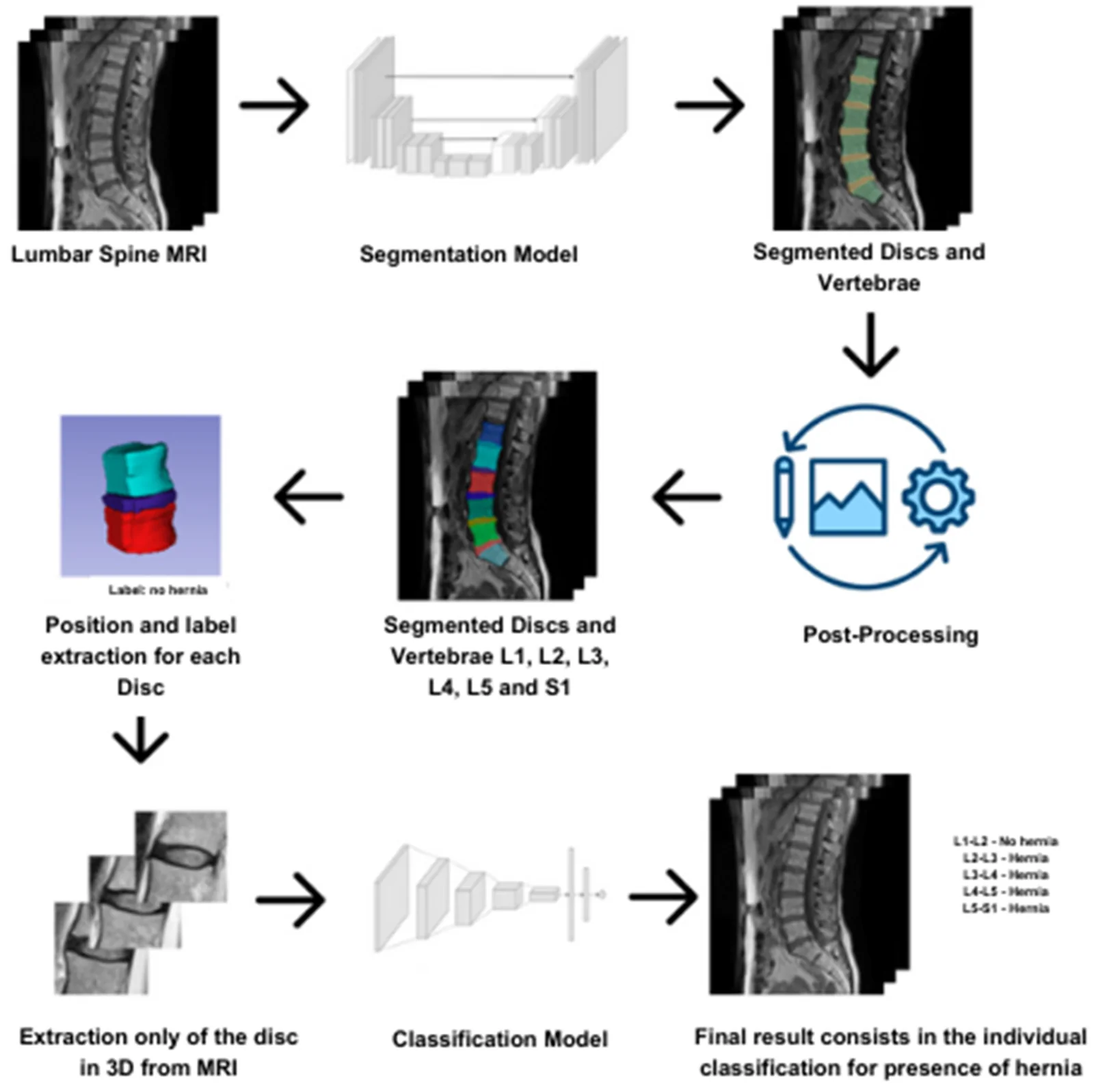
Description of the structured deep learning pipeline general process applied for hernia detection.

A detailed demographic analysis of the dataset revealed that 60.3% of the MRIs were from male patients. The average age was 51.54 ± 16.2 years, with a range of 19 to 82 years. The average patient height was 1.757 ± 0.07 meters, ranging from 1.60 to 1.90 meters. The average weight was 84.20 ± 11.69 kilograms, with a range of 60 to 129 kilograms.

To facilitate model development, the dataset was further filtered to include only sagittal plane images, resulting in 325 series and 5,200 images for analysis. In total, 5,200 sagittal plane MRI images were used to train and validate the vertebrae and disc segmentation model. Due to limited sample sizes at individual lumbar levels—particularly at upper segments (L1–L2 and L2–L3)—performance metrics were not reported separately for each anatomical level. The classification model operated at the disc level and generated predictions for each lumbar segment (L1–L2 through L5–S1), which were directly compared against level-specific ground-truth annotations. However, to ensure statistical robustness, all disc-level predictions were pooled across lumbar levels when computing the final binary classification metrics, including the F1-score. All dataset splits were performed at the patient level to prevent data leakage between training and validation sets. The developed algorithm was trained and validated with the available 325 series. On average, the images presented 487.1 ± 121 lines in height and 486.35 ± 120 lines in width. There were no restrictions on the type of sequences used, but most of the sequences found in the studies were T1 and T2.

All dataset splits were performed strictly at the patient (MRI examination) level, ensuring that no slices, intervertebral discs, or derived image patches from the same case appeared in both the training and validation set.

### 2.2 Pre-processing

#### 2.2.1 Annotation + segmentation

Annotation was performed by five radiologists with experience levels ranging from 2 to 15 years. The sequences of the 165 cases analysed were presented as DICOM cases with non-standardized acquisition protocols. Only sagittal T2 and T1 turbo spin echo series, and axial T2 turbo spin series, were used for annotation. These specific MRI sequences were chosen because they provide optimal contrast and anatomical detail for assessing lumbar spine pathology, particularly disc herniation and nerve root compression. Sagittal T2 and T1 turbo spin echo (TSE) sequences offer high-resolution visualization of intervertebral discs, vertebrae, and surrounding soft tissues, allowing for accurate segmentation and classification of pathological changes. Axial T2 TSE sequences are crucial for evaluating the transverse plane, enabling precise identification of nerve root involvement and compression severity. By focusing on these standardized sequences, the algorithm development ensures consistency in image quality and diagnostic reliability across different MRI manufacturers, facilitating robust and generalizable deep learning models.

The cases were annotated using a per-segment approach and a questionnaire exactly developed for the project and aligned with evaluation systems currently used for degenerative spine diseases. Specifically, theMRI of each case with lumbar spine pathology was analysed across five segments: L1–L2, L2–L3, L3–L4, L4–L5, and L5–S1. For each segment, radiologists examined for the presence or absence of disc herniation. For Disc Displacement (21–23) the following classification system was applied:

- Absent – Negative
- Bulging – Present
- Protrusion – Present
- Extrusion – Present
- Sequestration – Present

Detailed segmentation process instructions were created for the team of radiologists to minimize errors. Before starting the segmentation process, each radiologist practiced, under supervision, with two clinical cases, which were subsequently analysed and reviewed. This review was conducted radiologists with 15 years of experience in radiological analysis, followed by feedback. Disc morphology was labelled as “present” for any structural abnormality (bulging, protrusion, extrusion, sequestration) and “absent” when radiologically negative. Although bulging may reflect age-related changes, it represents a measurable deviation from normal disc anatomy. Herniations involve more focal and clinically significant displacement. From an imaging perspective, both indicate non-normal findings, justifying their inclusion under a unified “present” category to ensure diagnostic consistency and algorithmic sensitivity.

The same group of five radiologists performed the initial segmentation of the spinal images to train the segmentation model with 3D Slicer Sagittal T1 and T2 MRI series were used.

Each series was separately loaded onto the 3D Slicer in DICOM format. Each segment (i.e., anatomical structure) was manually outlined using the “Segment Editor” module on each slice where their anatomical structures were visible.

For sagittal series, the segmentation involved approximately 11 slices per disc level. The following segments were marked for each sagittal slice:

1. L1 body
2. L2 body
3. L3 body
4. L4 body
5. L5 body
6. S1 body
7. L1–L2 disc
8. L2–L3 disc
9. L3–L4 disc
10. L4–L5 disc
11. L5–S1 disc

#### 2.2.2 Vertebral body segmentation

The vertebral body segment includes endplates, osteophytes, and anterior and posterior cortices. Following this description, an example segmentation of the vertebral bodies from L1–S1 in a midsagittal section in T2 is presented within the “Segment Editor” module (Figure 2).

**Figure 2:**
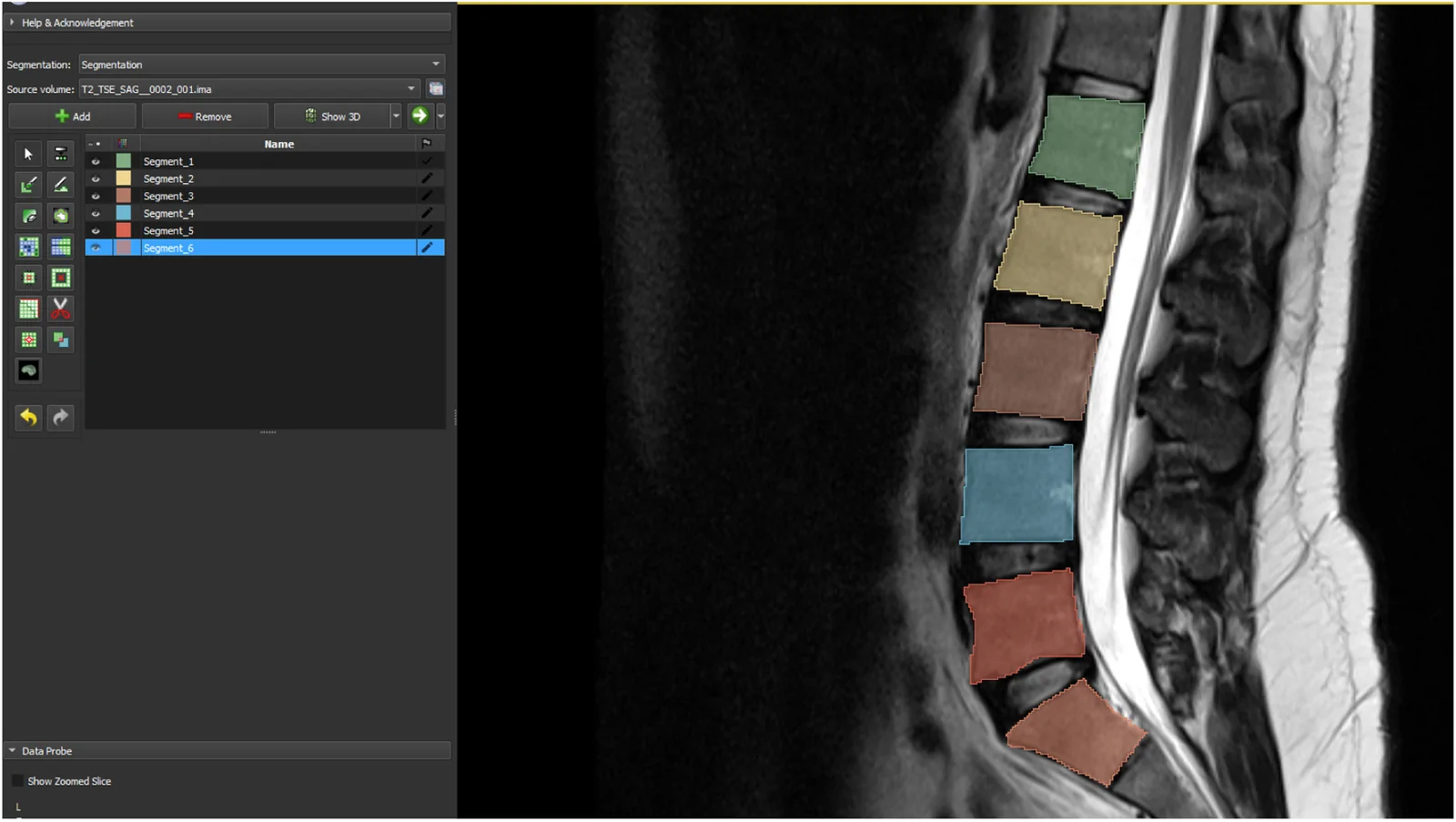
Example of vertebral body segmentation from L1–S1 in a Mid-sagittal T2 section, using the “Segment Editor” module.

The intervertebral disc segmentation includes protrusions, hernias, sequestrations, and Schmorl's nodes. The vertebral canal encompassed the cerebrospinal fluid space within the dural sac, as well as the epidural fat. The superior vertebral body of L1 was considered the cranial boundary, while the inferior endplate of S1 served as the caudal boundary. The lateral recesses were included in the vertebral canal segmentation. The manual segmentation of each case took about 90 min, and the analyses was extended to the above mentioned 5,200 images. When all the sagittal T2 slices were segmented, a configuration like the one presented in Figure 3 was obtained. Following the segmentation stage, a dedicated post-processing module was applied to refine the predicted disc regions, suppress artefacts, and enforce anatomical consistency across adjacent slices. The structure and workflow of the post-processing system implemented in the proposed framework are illustrated in Figure 4.

**Figure 3:**
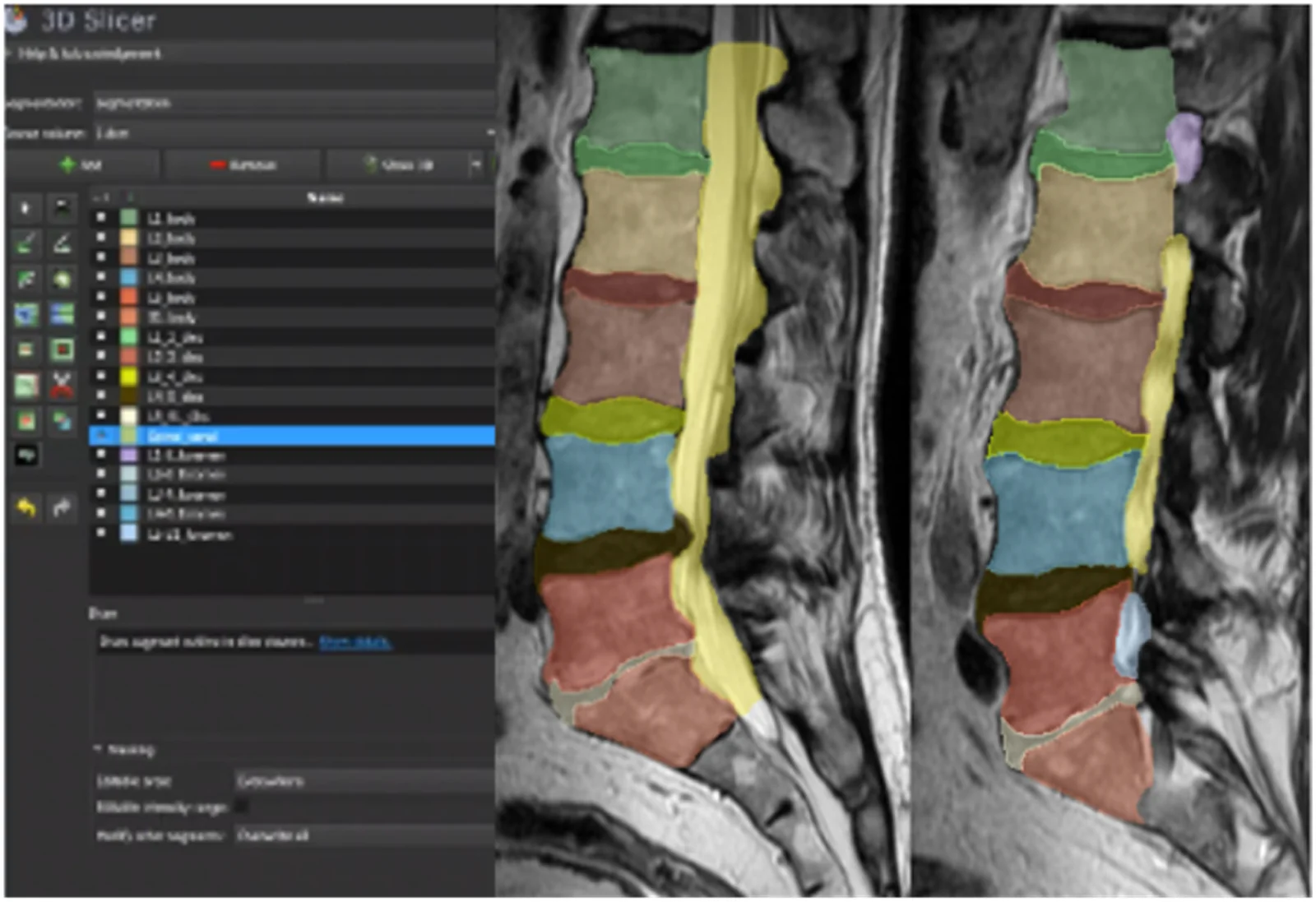
Example of the appearance of a spine after segmentation of sagittal T2 slices.

**Figure 4:**
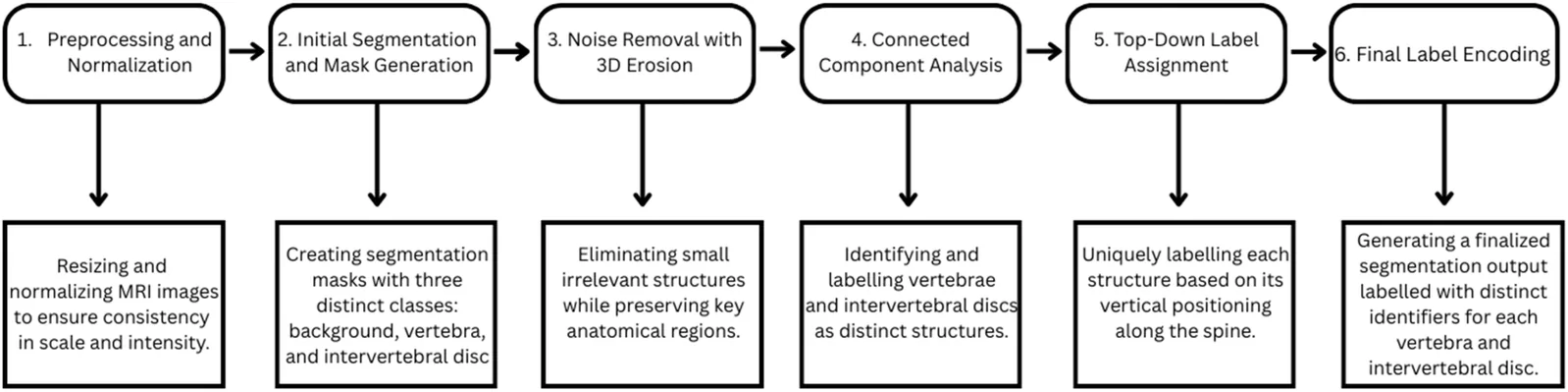
Steps of the post-processing system applied.

### 2.3 Final approach

The application of a single multi-label classifier for hernia detection would be suboptimal due to the complexity of lumbar spine anatomy and the significant anatomical variability among patients. To address this limitation, a structured deep learning pipeline was implemented in three stages, progressively refining feature extraction before classification. Figure 1 illustrates the general process applied.

Firstly, a segmentation model was trained to distinguish intervertebral discs, vertebrae, and background in lumbar MRI images. However, this model alone does not differentiate between individual discs (e.g., L1–L2 vs. L2–L3) nor eliminate spurious predictions. To address this, a post-processing step was introduced to refine the 3D segmentation masks, assign correct anatomical labels to each disc and vertebra, and remove hallucinatory pixels or outliers. Then, using the refined segmentation masks, individual 3D patches of the discs were extracted from the original MRI images. These were used to train a 3D classifier to determine whether each disc is herniated or not. This end-to-end pipeline enables automated, structured, and anatomically precise classification of hernias directly from lumbar MRI images.

For the multiclass segmentation task, we conducted an extensive evaluation of various encoder-decoder architecture combinations. The encoders tested included ResNet34, EfficientNet-B2, VGG16, and Mix ViT (24–27), while the decoders comprised UNet (28), UNet++ (29), DeepLabV3+ (30), SegFormer (31), and MANet (32). Each encoder was paired with each decoder to assess performance across different architectural configurations. The input images were resized to a fixed resolution of 256 × 256 pixels, and a batch size of 4 was used throughout training. Optimization was performed using the Adam optimizer (33) with a learning rate set to 1e-4. To address the inherent class imbalance in the segmentation task, the Dice Loss function was employed, which is particularly effective in evaluating overlap between predicted and ground truth masks.

### 2.4 Disc and vertebra segmentation from lumbar MRI

The segmentation of intervertebral discs and vertebrae from lumbar MRI images was performed using deep learning-based segmentation models. Several encoder-decoder architectures were applied to evaluate performance on this segmentation task. The models and parameters used are described in Section 2.3. The models were trained and validated using a custom dataset, divided into training and validation sets to ensure a fair evaluation among different architectures.

### 2.5 Dataset preparation

The dataset used for training and validation consisted of sagittal lumbar spine MRI images. Prior to training, the data were pre-processed to normalize pixel intensities and resized to dimensions of 256 × 256 pixels, ensuring compatibility among different model architectures. The dataset split was as follows: 80% samples for training and 20% samples for validation.

Each dataset sample consisted of a grayscale MRI image and its corresponding ground truth segmentation mask. The masks contained three classes: background, intervertebral discs, and vertebrae. These three classes were extracted from the annotations provided by radiologists, with classes 1 to 6 belonging to vertebrae and classes 7 to 11 representing discs. Due to low sampling (limited cases), the application of one neural network model was insufficient for dividing all vertebral bodies. Thus, a simpler classification between vertebrae and discs was adopted, and image post-processing techniques were utilized to regain the 1-to-11 classification. Since the primary focus was on discs, their extraction became essential for accurate disc-level classification results.

Several encoder-decoder architectures were tested for segmentation, combining various encoders (previously named) with U-Net, U-Net++, DeepLabV3+, SegFormer, and MAnet. The models used a batch size of 8 and a learning rate of 1e-3 was chosen. The Adam optimizer was used, and the Dice Loss function for multi-class segmentation was applied to optimize model performance. To ensure unbiased comparison, the same optimizer and hyperparameters were used across all experiments.

Each model underwent 30 epochs of training, using a learning rate scheduler called ReduceLROnPlateau, designed to dynamically adjust learning conditions throughout training. During evaluation, the Dice Coefficient was adopted as the primary metric, aiming to quantify the overlap between the predicted masks and the ground truth masks. This metric is widely recognized for its effectiveness in assessing segmentation models, particularly in contexts that demand high precision in matching predicted data to real data.

When evaluating the performance of image segmentation architectures, such as U-Net, it is critical to use evaluation metrics that accurately represent the quality of segmentations generated by the network. The appropriate choice of these metrics significantly impacts the validation and comparison of different models.

After segmenting intervertebral discs and vertebrae using the best deep learning-based model trained, a post-processing procedure was applied to assign anatomical labels to the segmented structures. While the segmentation model predicts three classes—background, vertebra, and disc—it does not inherently distinguish individual vertebrae or specific intervertebral discs.

Therefore, a post-processing system was implemented to improve segmentation results, eliminate noise, and accurately label each vertebra and disc based on its anatomical position.

The post-processing involved the following steps:

### 2.6 Herniated disc classification using 3D patch extraction

Following the segmentation of intervertebral discs and lumbar vertebrae using the best model trained in the previous section, a second model was trained to classify whether each intervertebral disc presented a hernia. This classification model uses 3D patches extracted from the original MRI images, based on segmentation masks obtained.

The dataset for training and validation in this classification task was derived directly from the segmentation dataset. The same train-validation split was maintained for consistent experimentation. Each intervertebral disc was localized using its corresponding segmentation mask. The centre of each segmented disc was determined, and a 3D patch around this centre was extracted from the original MRI image. For cases where the extracted region contained fewer slices than required, zero-padding was applied to standardize input shapes. Each extracted patch was labelled as herniated or non-herniated based on a manually curated dataset.

To improve the model's generalization capabilities, several data augmentation techniques were applied to the extracted 3D patches:

- Gaussian Noise: Introduces slight variations in pixel intensities.
- Random Brightness and Contrast: Simulates variations in intensity levels.
- Random Motion Artifacts: Replicates motion-induced distortions common in MRI scans.
- Random Affine Transformations: Introduces small rotations and scaling.
- Elastic Deformations: Aids in generalizing to subtle anatomical variations.

These techniques were implemented using the *TorchIO* library, specialized for 3D medical images.

For classification, various 3D deep learning architectures were evaluated for their performance in predicting herniated discs. These models are widely used in medical imaging due to their ability to capture spatial dependencies in volumetric data.

For the binary classification task, we evaluated a range of 3D deep learning architectures, including 3D ResNet-50, 3D EfficientNet-B2, 3D SEResNeXt-50, 3D SEResNet-50, and a 3D Vision Transformer (ViT). We used it from Monai open source Python's module (ref: https://monai.io/). All models received input volumes of size (20, 80, 80), corresponding to the depth, height, and width dimensions, respectively. A batch size of 4 was used during training to accommodate the high memory demands of 3D data. The models were optimized using the Adam optimizer, and the loss function employed was the Binary Cross-Entropy with Logits Loss (BCEWithLogitsLoss), suitable for binary classification in neural networks.

The classification task was framed as a binary problem, where the model's output is a probability score. A threshold of 0.5 was used to determine whether a disc was classified as herniated, denoted by 1, or non-herniated, denoted by 0.

To ensure comprehensive analysis, both convolution-based models (CNNs) and transformer-based models (ViT) were tested in the 3D domain. Each architecture was trained and evaluated using the same dataset split and preprocessing pipeline. By maintaining identical training conditions, any performance differences were attributable to the model architectures rather than hyperparameter variations. The evaluation metrics included Accuracy, AUCand F1-Score. However, due to class imbalance in the dataset, F1-Score was chosen as the primary metric.

## 3 Results

The following sections present the results obtained during different phases of the proposed pipeline: initial segmentation, post-processing, and classification.

### 3.1 Pre-annotation and final segmentation

Following the first round of annotations, 51 exams were segmented across 18 established classes, with 17 corresponding to anatomical structures and one reserved for the background. Due to the limited number of available exams at this initial stage, a segmentation model was trained to operate on sagittal slices and incorporate all classes −17 manually segmented classes plus the background.

The chosen architecture was a U-Net with an EfficientNet-B2 backbone, achieving a Dice Score of 0.734. The reported mean Dice score corresponds to the macro-average across segmentation classes, computed over all cases in the evaluation set. Extensive experiments were not conducted at this stage since the goal was to develop an initial model capable of reasonably accurate pre-annotation to expedite and ease the workload of radiologists. It is worth emphasizing that one of the primary barriers to adopting AI solutions in healthcare is the reliance on specialized, high-precision annotations (11).

Using this initial model, the remaining 114 exams were automatically pre-annotated. During this phase, smaller structures, such as foramina, demonstrated inferior segmentation performance compared to larger structures, like vertebral bodies. All exams were subsequently reviewed and corrected by radiologists, resulting in a validated final dataset that served as the foundation for subsequent stages.

Despite reaching a total of 165 exams and over 5,200 annotated sagittal images, attempts to train a new batch of segmentation models did not exceed an F1-Score of 0.765. Classes with lower sample sizes continued to exhibit the weakest metrics. Furthermore, radiologists annotated various pathological characteristics for each case, yielding up to 65 classes per patient, including 13 potential attributes for each intervertebral disc. However, the low sample variability rendered the training of a robust model for this multiclass task impractical.

Given these circumstances, it was decided to narrow the segmentation objective and focus on a classification task with greater learning potential within the available sampling. The problem was reformulated into segmentation with only three classes: background (class 0), vertebrae (class 1), and discs (class 2). This simplification increased the sample density per class and potentially improved the metrics obtained, serving as the foundation for subsequent pipeline steps.

In this new experimentation, the same 5,200 sagittal images were re-annotated with only three classes. Due to sample limitations per series, 3D models were avoided, and tests focused on 2D architectures. The segmentation models evaluated are detailed in the methods section of the segmentation task.

To ensure a fair comparison among architectures, all were trained on the same dataset, utilizing the same loss function and hyperparameters, such as the learning rate. Each training session lasted between 1 and 3 min, with experiments conducted for 30 epochs, totalling less than two hours of training per model. The results are presented in Table 1.

**Table 1:** Results for the segmentation model each decoder was trained with each encoder to achieve the best possible model for the task.

Segmentation model (Mean Dice Score)	ResNet34	EfficientNet-B2	VGG16	Mix ViT
U-Net	0.776	0.897	0.895	0.769
U-Net++	0.883	0.892	0.771	*
DeepLabV3+	0.878	0.890	*	0.875
SegFormer	0.887	0.889	0.885	0.757
MAnet	0.876	0.898	0.768	

The metric used was the Mean Dice Score (Macro Dice Score) across all model classes.

The asterisks (*) denote the encoders that were not compatible with the decoders used in these scenarios.

Notably, EfficientNet-B2 demonstrated consistent performance across different architectures, with MAnet achieving the highest Mean Dice Score of 0.898. The reported mean Dice score corresponds to the macro-average across segmentation classes, computed over all cases in the evaluation set. This result suggests that hybrid architectures, which combine convolutional mechanisms with attention mechanisms, were more effective than purely convolutional or purely attention-based models for medical segmentation tasks, highlighting promising directions for future research.

With this selected architecture, new masks were generated for all images in the dataset, replacing manual annotations with model predictions. Figure 5 illustrates an example of a mask generated on a sagittal slice, comparing it to the original manual segmentation consisting of 18 classes.

**Figure 5:**
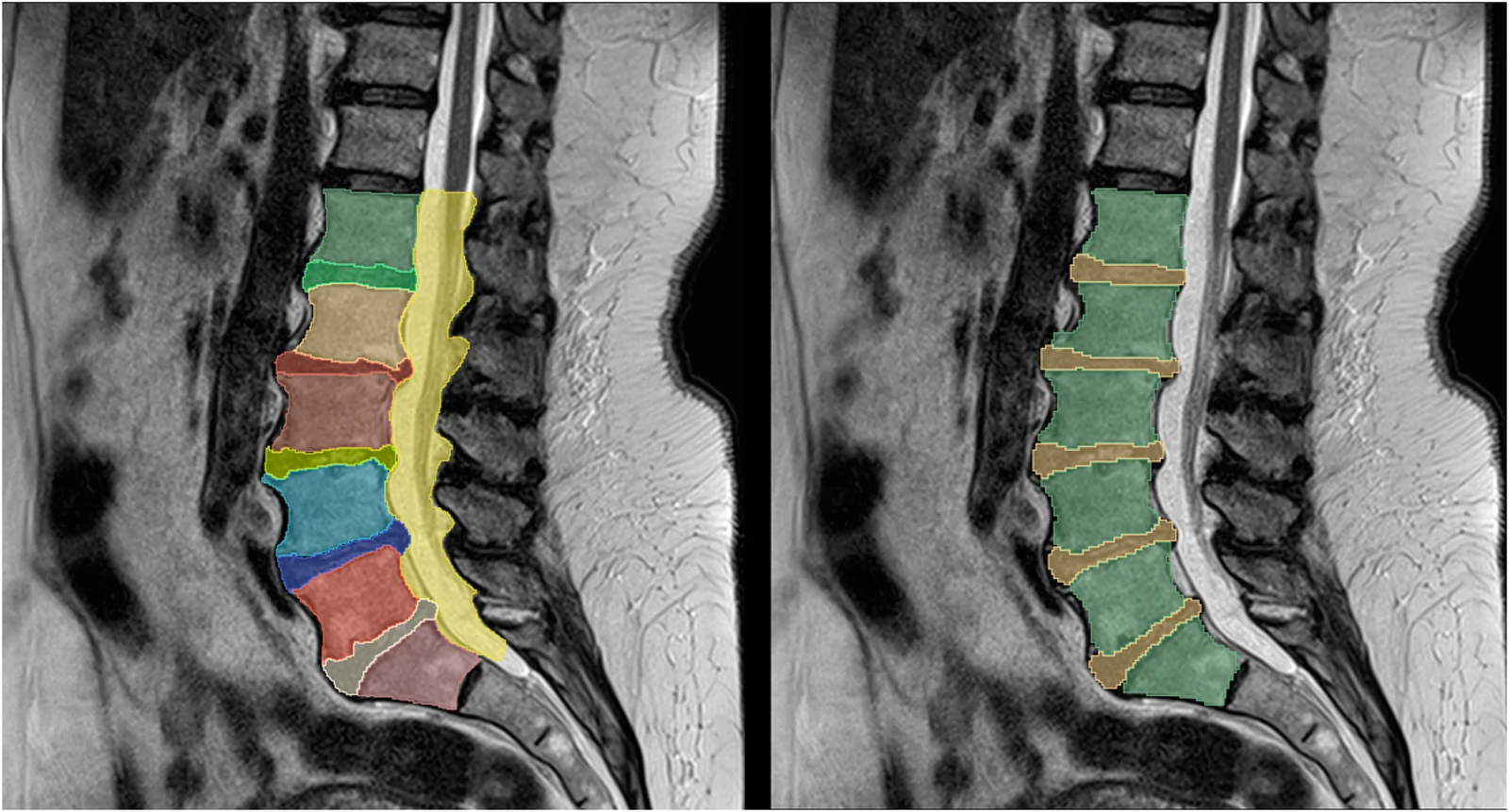
Segmented mask created by radiologists with 18 classes (left) compared to the mask segmented by the trained model with 3 classes (right).

From this new database, automatically segmented into three classes (background, vertebra, and disc), training and validation sets were structured for classification. It is acknowledged that this procedure could introduce bias, as the same model was utilized to generate the masks that feed the classifier. However, the objective was to realistically simulate a fully automated inference pipeline, where the classifier operates based on segmentations previously generated by AI. To mitigate risks of data leakage and bias, it was ensured that the exams used in the segmentation validation set were also used for validation during classifier training.

### 3.2 Post-processing

The limitation of the segmentation model in distinguishing only three classes (background, vertebrae, and discs) provided greater precision in volumetric structure segmentation but lost anatomical granularity. Specifically, it could not individually identify each vertebra (e.g., L1, L2, etc.) or each intervertebral disc (e.g., L1–L2, L2–L3, etc.). This differentiation is crucial for the subsequent classification stage, as the classifier models must operate on specific discs.

While it would have been possible to train a segmentation model with a multi-class output consisting of at least 12 distinct classes (6 vertebrae, 5 discs, and background), this added complexity; combined with the limited available sample size; would compromise the model's performance, as evidenced by the drop in Dice Score during initial attempts.

Given this scenario, a post-processing pipeline was implemented for the segmentation masks generated by the three-class model. This pipeline focused on separating the segmented structures along the Z-axis enabling the individualization of anatomical volumes. Small volumes identified as noise were removed using morphological thresholds, contributing to refined masks and enhanced fidelity in the representation of real anatomical structures. Figure 6 illustrates this process and the effects of post-processing on the generated masks.

**Figure 6:**
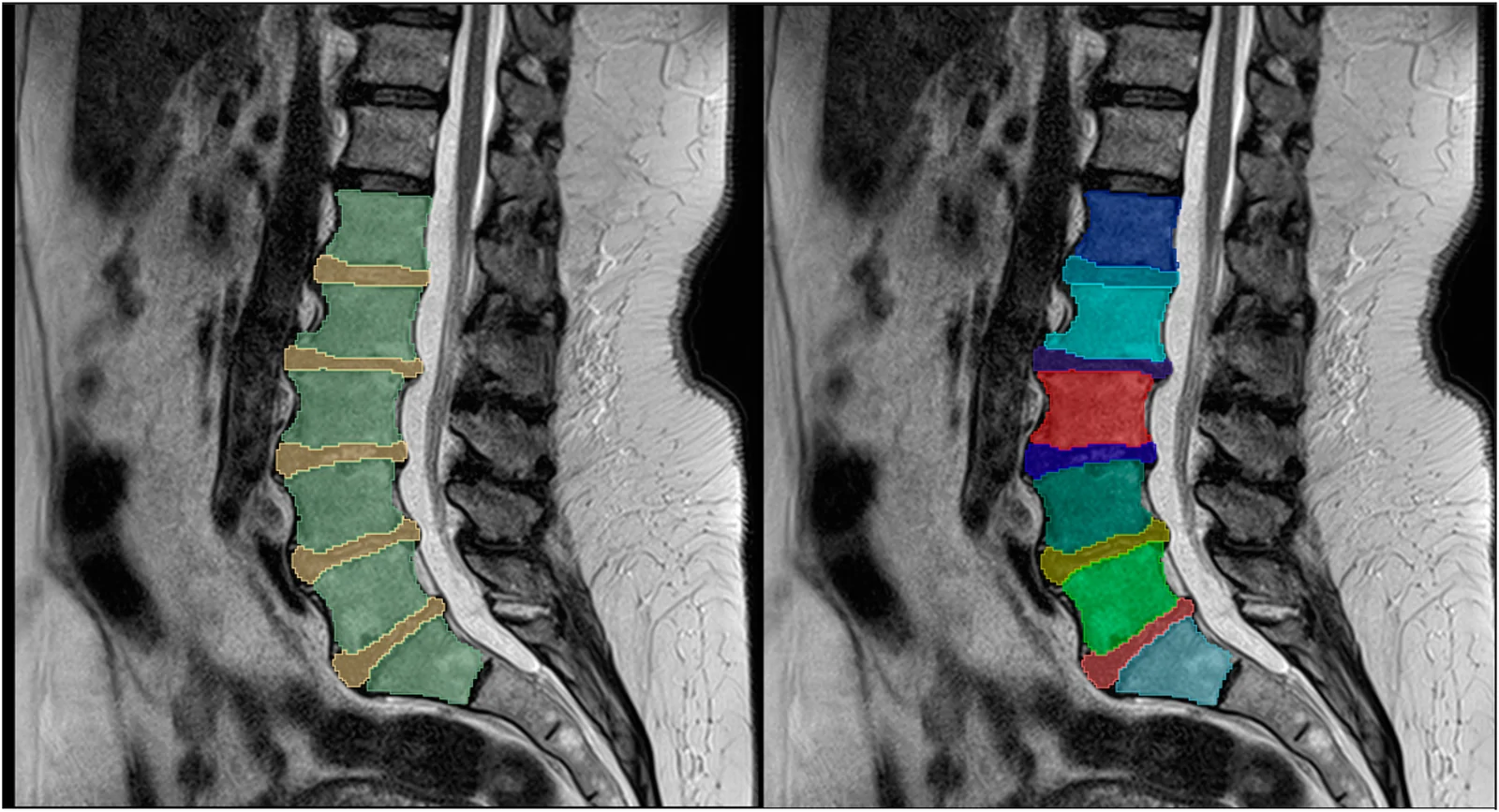
Mask generated by the three-class segmentation model (left) and the same mask post-processed to independently extract each disc and vertebra (L1, L2, etc.) (right).

### 3.3 Classification model

With each intervertebral disc individually identified through segmentation and post-processing, it became possible to extract the geometric centre of each disc volume. This centre served as the basis for cropping the volume used in the classification task. From the central pixel of the disc, a volumetric window was extracted and used as input for the classification models.

The training for the classification stage took between 30 min and 1 h, depending on the tested architecture, with all being evaluated over 30 epochs. The four 3D models described in the methodology were compared using identical hyperparameters to ensure fairness in the analysis. The results for F1-Score, AUC and accuracy on the validation set are presented in Table 2. The best performance achieved was an F1-Score of 0.72, an AUC of 0.82 and an accuracy of 0.71.

**Table 2:** Performance of classification models trained on vertebral volumes extracted from segmentation outputs.

Herniation classification	F1-Score	Accuracy	AUC
ResNet18	**0**.**72**	**0**.**71**	**0**.**82**
EfficientNet-B2	0.66	0.67	0.71
SENet (ResNet50)	0.71	0.68	0
ViT	0.71	0.73	0.8

All models were trained using identical hyperparameters. The results highlight the intrinsic difficulty of the task and the high variability of the data, leading to performance levels consistent with underfitting, particularly for EfficientNet-B2 and the Vision Transformer (ViT).

Bold values indicate the highest performance achieved for each metric across the evaluated models.

After intervertebral disc segmentation and extraction of disc-centered volumes, classification models were trained for binary disc herniation detection. A fixed set of 100 MRI studies was reserved for final validation, with all five lumbar discs evaluated per patient. This fixed cohort constitutes a single, independent patient-level hold-out test set, used exclusively for final model evaluation. No cross-validation or resampling was performed on this test set. As summarized in Table 2, all models achieved moderate performance, with F1-scores ranging from 0.66 to 0.73. Overall, the results indicate a consistent pattern of underfitting across architectures, particularly for EfficientNet-B2 and the Vision Transformer, characterized by limited performance on both training and validation sets despite identical hyperparameter configurations.

The F1-Score was selected as the primary evaluation metric due to its robustness in scenarios with imbalanced classes—a common characteristic of medical datasets. The F1-Score represents the harmonic mean between precision (the proportion of correct predictions among positives) and recall (the ability to capture all true positives). The fact that the F1-Score exceeded the accuracy indicates that the model was more effective in identifying discs with abnormalities, even with a slightly lower overall accuracy due to the high number of negatives (normal discs).

This behaviour was deliberately calibrated using a loss function composed of the average between Binary Cross-Entropy (BCE) and F1 Loss. It was observed that using BCE exclusively resulted in higher accuracy but led to a drop in the F1-Score.

The results suggest intrinsic limitations in the data rather than architectural shortcomings. Three main factors likely contributed to this outcome: (i) the relatively small number of annotated samples per class, (ii) high intra- and inter-patient variability, including examinations acquired from multiple vendors and protocols, and (iii) annotation uncertainty, as labels were originally assigned by different radiologists and remained subjectively challenging even after standardization. In addition, borderline clinical cases—such as mild disc bulging in elderly patients—are often interpreted in conjunction with broader clinical context, which is unavailable to the model given the isolated disc-centered input volumes.

## 4 Discussion

The adoption of a multi-stage pipeline - segmentation, post-processing, and subsequent classification -was a strategic choice to address the inherent challenges of applying deep neural networks to the detection of lumbar hernias. Attempting to train a direct 3D model to classify intervertebral discs with multiple classes (e.g., three positive and two negative classes per volume) would have resulted in a problem of high ambiguity. In this scenario, the model would receive a volume containing several discs with different pathological states but have limited contextual cues to determine which anatomical regions correspond to each class. Such an approach would likely compromise model accuracy, especially with the limited dataset sampling available in this study (12, 13).

Several deep learning–based approaches have previously been proposed for the automated detection of lumbar disc herniation and degenerative spine pathology from MRI, demonstrating the feasibility of AI-assisted spinal imaging (11–13). However, direct quantitative comparison across studies remains challenging due to heterogeneity in datasets, annotation strategies, pathological definitions, and evaluation protocols. Many reported methods perform slice-level or volume-level classification, which can introduce ambiguity when multiple intervertebral discs with different pathological states coexist within a single examination (12, 14). In contrast, the proposed approach adopts a disc-centric strategy by explicitly segmenting vertebrae and intervertebral discs, followed by anatomically informed post-processing prior to classification. This design improves anatomical interpretability and aligns more closely with clinical reporting practices.

Another distinguishing feature of the present work is the use of a structured, multi-stage pipeline integrating segmentation, post-processing, and classification. While end-to-end classification models have been reported, they often lack explicit anatomical localization, limiting interpretability and robustness (15, 16). The proposed approach addresses this limitation while also enabling scalable annotation and dataset expansion, which remains a significant bottleneck in medical imaging research (9, 17). In addition, the use of multi-vendor MRI data introduces realistic inter-scanner variability, enhancing translational relevance compared to studies relying on single-vendor datasets, albeit at the cost of increased task complexity (15). Finally, this study provides a controlled comparison of convolutional and transformer-based architectures under identical experimental conditions, addressing a gap in the current literature where architectural comparisons are often confounded by experimental variability (18–20).

Despite these merits, important limitations remain, particularly related to dataset size, class imbalance, and data heterogeneity, which affected classification performance and led to a consistent pattern of underfitting across architectures. Although the proposed pipeline enabled anatomically grounded disc-level classification, all evaluated models achieved only moderate performance, reflecting limited generalization under the available training data. These constraints motivated the focus on binary disc-level classification in the present study, with future work directed toward dataset expansion, improved annotation consistency, and external validation.

The strategy of segmenting the discs first enabled a crucial spatial refinement: by isolating the volume of each disc, it became possible to accurately associate each sample with its respective annotation, eliminating the need for the model to “figure out” the disc locations during classification. This approach not only reduced noise in the classifier inputs but also significantly increased the interpretability of the results (14, 15). Because disc-level classification was performed exclusively on regions extracted from automatically generated segmentation masks, segmentation errors may propagate to the classification stage. Quantifying this effect through direct comparison with ground-truth masks was not feasible due to limited manual annotations and is an important direction for future work.

This concern with the explicit anatomical localization of discs has been emphasized in recent studies and competitions focused on the detection of lumbar spine degeneration, provided data that included not only degeneration classes by disc but also the central coordinates (X and Y) of each disc in the image, underscoring the importance of precise localization for this task. The methodology developed in this study automates this extraction process from the segmented masks, facilitating not only current classification efforts but also enabling future dataset expansion (16). Once the discs are segmented and localized, it becomes possible to add new annotations directly at the disc level without the need to review the entire series slice by slice, making the annotation process more scalable and less labor-intensive for experts (17).

This capability to automatically generate the anatomical centres of discs from segmentation also represents a significant methodological contribution. By structuring the data in this manner, the proposed pipeline establishes a solid foundation for constructing enriched datasets that can be utilized in supervised models with greater granularity and reliability. Furthermore, this structuring paves the way for future applications using multimodal models that combine spatial information with additional clinical or radiological data (18).

### 4.1 Results and insights

The results obtained in this project demonstrate that even with relatively limited sampling, it was possible to achieve significant metrics in both segmentation and classification. All classification performance metrics reported in this section were computed exclusively on the fixed patient-level hold-out test set comprising 100 MRI examinations. The segmentation model achieved a Dice Score of 0.898 with only three classes, providing a robust foundation for the subsequent classification stage. Hence, it is proposed that future annotation cycles prioritize classification tasks since the segmentation stage can now be automated with high precision, significantly reducing the human effort required (19).

Another noteworthy aspect of this study was the ability to systematically compare different neural network architectures for the same task in both segmentation and classification stages. The standardization of hyperparameters, loss functions, and datasets used allowed for a fair and direct analysis of each model's generalization capacity, isolating the impact of the architecture itself. This approach is particularly relevant in the field of deep learning for medical imaging, where variations in data or training parameters can introduce significant biases in performance evaluation (20).

### 4.2 Architectural comparisons

In segmentation, for example, it was observed how different architectures handle the morphological complexity of anatomical structures, particularly in the context of limited sampling. Purely convolutional models, such as the traditional U-Net, demonstrated consistent performance but fell short compared to models incorporating more advanced mechanisms for capturing global context (34). On the other hand, attention-based models, such as Vision Transformers (ViT), struggled to capture fine anatomical details, especially for smaller structures (34, 35).

The most notable result was achieved with the MA-Net using EfficientNet-B2 as its backbone—a hybrid architecture combining deep convolutions with attention-inspired mechanisms (36). This performance suggests that, for medical segmentation tasks, architectures that balance the spatial robustness of convolutions with the contextual capacity of attention mechanisms may offer significant advantages. This observation opens avenues for future investigations into hybrid models optimized specifically for the medical domain, where the balance between local detail and global context is essential (34–36).

In contrast, for the disc-level binary classification task, the best performance was obtained with a ResNet18 backbone. This finding suggests that, once anatomical localization and spatial normalization are handled by the preceding segmentation and post-processing stages, smaller and less complex architectures may be sufficient—or even preferable—for classification. Under these conditions, the classifier operates on isolated disc-centered volumes with reduced structural variability, diminishing the need for deeper or attention-heavy models and favoring architectures with lower capacity and improved generalization.

Furthermore, while the current classification performance remains limited under the available data regime, the strong results achieved in segmentation and disc extraction indicate that the main bottleneck lies in data scale and annotation consistency rather than in the pipeline design itself. As such, expanding the dataset and validating the approach on independent, multi-institutional cohorts are expected to be critical steps toward improving disc-level classification performance and assessing clinical applicability.

### 4.3 Implications and limitations

The richness of this inter-architectural comparison contributes not only to the selection of the most effective model in this study but also provides a methodological basis for researchers facing similar challenges. In a field with a multiplicity of models and the continuous emergence of new architectures, controlled comparisons like this offer practical guidelines and empirical evidence to inform model selection for specific tasks in healthcare (18).

However, one of the main limitations of this study lies in the class imbalance, particularly among the positive classes of disc alterations. Under these conditions, all evaluated classifiers exhibited moderate performance and a consistent pattern of underfitting, limiting their ability to generalize beyond coarse binary discrimination. This constraint makes binary disc-level classification a more feasible solution at this stage. Additionally, the absence of an external test set prevents the definitive validation of the generalization capability of the developed models (37). Future research should focus on creating an external validation set, ideally with data from other institutions, to ensure greater robustness and clinical applicability.

It is also worth noting that the limited sampling is related to the depth of the annotations performed. This work involved annotations across multiple anatomical and pathological levels, which required considerable expert effort, making the dataset construction extremely labor-intensive. As a result, it was not possible to fully explore the annotated dataset's potential, such as the 12 distinct classes at the disc level or other anatomical structures beyond discs and vertebrae. Nevertheless, this wealth of annotations provides the dataset with high scientific value, allowing for reuse in future research. The annotation granularity achieved in this study aligns with gold standards in international literature, contributing to the development of more interpretable and clinically relevant AI models.

Importantly, while qualitative visualization of classifier outputs (e.g., true positive and false negative examples overlaid on MRI scans) can be valuable for clinical interpretability, such illustrations were not included in the present study. The proposed classification operates on cropped 3D disc volumes derived from automatically generated segmentation masks rather than on full-field MRI slices, making direct visualization on original images non-trivial and potentially misleading without additional post-processing and expert curation. Moreover, selecting representative cases for visualization may introduce subjective bias and may not accurately reflect overall model behavior across heterogeneous acquisition protocols. The present work therefore prioritizes systematic quantitative evaluation of the fully automated pipeline. The development of standardized visualization tools and clinically interpretable output overlays is an important direction for future work, particularly in the context of prospective validation and clinical deployment.

This study not only presents a viable pipeline for the partial automation of medical imaging tasks but also serves as a foundation for future developments, both in clinical applications and scientific investigations.

In the medium to long term, future research should aim to extend the current AI pipeline by incorporating multimodal data sources, such as clinical reports, patient histories, and biomechanical data, into integrative models that move beyond image-based classification (38). Such multimodal frameworks could significantly enhance diagnostic accuracy and enable personalized treatment planning. Additionally, expanding the dataset with more diverse samples from multiple institutions will be critical to improving model generalizability and addressing current limitations due to class imbalance and sampling bias and size. From a methodological standpoint, the integration of spatiotemporal models and recurrent transformer architectures could allow for longitudinal analysis of degenerative progression, offering predictive capabilities that inform early interventions. Furthermore, developing explainable AI (XAI) mechanisms tailored to spine diagnostics would increase clinical trust and facilitate regulatory approval (39). Finally, real-world deployment studies in radiology workflows are essential to evaluate usability, cost-effectiveness, and impact on patient outcomes, thereby closing the translational gap between algorithm development and clinical practice.

## Data Availability

The raw data supporting the conclusions of this article will be made available by the authors, without undue reservation.
